# Nutritional Aspects in Diabetic CKD Patients on Tertiary Care

**DOI:** 10.3390/medicina55080427

**Published:** 2019-08-01

**Authors:** Claudia D’Alessandro, Massimiliano Barsotti, Caterina Cianchi, Claudia Mannucci, Riccardo Morganti, Serena Tassi, Adamasco Cupisti

**Affiliations:** 1 Department of Clinical and Experimental Medicine, University of Pisa, 56126 Pisa, Italy; 2 Nephrology, Transplant and Dialysis Unit, University Hospital of Pisa, 56126 Pisa, Italy; 3 Section of Statistics, University Hospital of Pisa, 56126 Pisa, Italy

**Keywords:** CKD, physical performance, functional capacity, diet, nutrition, protein intake, body composition, diabetes, diabetic nephropathy

## Abstract

*Background and objectives*: Diabetes is largely prevalent in the chronic kidney disease (CKD) population. Both conditions have metabolic and nutritional abnormalities that affect body composition and the presence of diabetes makes the dietary management of CKD patients more difficult. The aim of this study was to assess peculiar nutritional and functional aspects of diabetic patients in an adult/elderly CKD population, and their predictive significance. *Materials and methods*: This prospective cohort study included 144 out-patients aged >55 years, affected by stage 3b-4 CKD, on tertiary care clinic; 48 (40 males) were type 2 diabetics and 96 (80 males) were nondiabetics. The two groups have similar age, gender, and residual renal function (30 ± 9 vs. 31 ± 11 mL/min×1.73). All patients underwent a comprehensive nutritional and functional assessment and were followed for 31 ± 14 months. *Results*: Diabetic CKD patients showed higher waist circumference and fat body mass, lower muscle mass, and lower number of steps per day and average daily METs. Meanwhile, resting energy expenditure (REE), as assessed by indirect calorimetry, and dietary energy intake were similar as well as hand-grip and 6 min walking test. Diabetic patients did not show a greater risk for all-cause mortality and renal death with respect to nondiabetics. Middle arm muscle circumference, phase angle, serum cholesterol, and serum albumin were negatively related to the risk of mortality and renal death after adjustment for eGFR. *Conclusions*: CKD diabetic patients differed from nondiabetics for a greater fat mass, lower muscle mass, and lower physical activity levels. This occurred at the same REE and dietary energy intake. The outcome of diabetic or nondiabetic CKD patients on tertiary care management was similar in terms of risk for mortality or renal death. Given the same residual renal function, low levels of muscle mass, phase angle, serum albumin, and cholesterol were predictive of poor outcome. Overall, a malnutrition phenotype represents a major predictor of poor outcome in diabetic and nondiabetic CKD patients.

## 1. Introduction

Chronic kidney disease (CKD) is prevalent in the elderly and it is often associated with cardiovascular risk, increasing prevalence of frailty, disability and malnutrition [1,2], and comorbid conditions. Among these, diabetes mellitus is largely prevalent and still increasing.

Both diabetes and CKD have metabolic and nutritional abnormalities that make dietary management quite difficult, especially in elderly patients with moderate to severe reduction of renal function [3].

Correct management of energy intake is essential to prevent the onset of protein energy wasting (PEW), especially on low-protein regimes. The prevalence of PEW in mild to moderate CKD patients ranged from 6 to 10% and is even higher in the elderly CKD patients [4].

In CKD patients, the scenario is even more complex due to the high prevalence of overweight or obese states. Obesity contributes to physical limitations and it is an independent risk factor for CKD and cardiovascular disease [5,6]. Therefore, obesity is another nutritional concern that may complicate the dietary management of advanced CKD patients.

Abnormalities in body composition and functional status are associated with poor quality of life, and increased risk of PEW, morbidity, and mortality [7].

In nondiabetic CKD patients, most of the current guidelines suggest an energy intake of 30 kcal/kg/d for subjects over 60 years of age, and 35 kcal/kg/d for subjects younger than 60 years. Normal to reduced protein (0.8–0.6 g/kg/d), sodium (100 mmol/d), and phosphorus (800–600 mg/d) intake are also recommended in order to correct metabolic and nutritional abnormalities and to prevent PEW [8].

Many uncertainties still exist in the management of the nutritional therapy of diabetic patients with CKD. In 2014, a consensus conference, including the American Diabetes Association, the American Society of Nephrology, and the National Kidney Foundation, outlined dietary recommendations for diabetic kidney patients [9]. In summary, the expert panels recommend a dietary protein intake of 0.8 g/kg of body weight per day for patients not on dialysis, carbohydrates from sugars less than 10% of energy intake, high fiber consumption, and higher polyunsaturated and monounsaturated fat intake (instead of saturated fatty acids, trans-fat, and cholesterol) that are associated with more favorable outcomes. They also recommend a dietary sodium restriction of less than 1.5–2.3 g/day to be individualized on the basis of patient’s needs [9].

A further concern is the high prevalence of sedentary lifestyle in the general population, including diabetics and renal patients. It has been shown that adults with hypertension and/or type II diabetes, overweight condition, and with other comorbidities do not reach an adequate physical activity level [10]. Sedentary habit plays a key role in the development of insulin resistance. For the elderly population with type II diabetes and/or hypertension, maintaining 30 min of moderate exercise five times per week or maintaining 150 min per week of physical activity is recommended to improve glycemic control and to maintain normal blood pressure levels [10,11]. Exercise training has beneficial effects on skeletal muscle and particularly on mitochondrial function and represents an important tool to prevent and to treat obesity and type 2 diabetes.

Sarcopenia, the term currently used to indicate reduced muscle mass and strength, is prevalent in CKD [12,13] and it is associated with increased mortality in patients with CKD [14,15]. An accelerated loss of muscle strength is observed in elderly patients with type 2 diabetes: insulin resistance, hyperglycemia, muscle fat infiltration, peripheral neuropathies, and oxidative stress are possible mechanisms [16].

The aim of this study was to assess peculiar nutritional and functional aspects of diabetic patients in an adult/elderly CKD population, and their predictive significance.

## 2. Materials and Methods

This is a prospective cohort study. It included 144 stable out-patients aged >55 years, affected by stage 3b–4 CKD, on tertiary care clinic; 48 (40 males) were type 2 diabetics and 96 (80 males) were nondiabetics. Patients were recruited from October 2014 to April 2018 and were followed up to April 2019. Ethical approval was obtained by the Ethics Committee of Pisa University Hospital on 18 November 2014 (study no. 409).

Standard care, including dietary support, was given to all the patients. Personalized nutritional approach consisted of a moderate protein restriction (0.6–0.8 g/day) and control of phosphate and sodium intake [17,18]. Patients were followed-up upon to record major outcomes such as mortality or renal death. Dialysis commencing or doubling serum creatinine were assumed as renal death. Causes of death were recorded in the hospital documents, whereas causes of death outside the hospital were reported by relatives. All patients underwent a comprehensive nutritional and functional assessment. It included urine and blood biochemistry, anthropometry and bio-impedance analysis, tests of physical activity and performance, assessment of dietary nutrient intake and of resting energy expenditure.

### 2.1. Biochemistry

Blood biochemical determinations included serum levels of creatinine, BUN, phosphorus, calcium, bicarbonate, albumin, PTH, and blood cell count. Urinary sodium, phosphate, and urea were measured in 24 h urine samples. Since all the patients were in stable conditions, protein catabolic rate (PCR) was considered as surrogate of the dietary protein intake, calculated by Maroni–Mitch’s formula [19] and normalized by body weight. All the determinations were performed with the methods routinely used by our lab. eGFR was calculated using the CKD-EPI formula [20].

### 2.2. Anthropometry

Height was measured with a stadiometer. Body weight was recorded with light clothes, without shoes on a mechanical scale. Body mass index (BMI) was calculated as body weight (kg)/height^2^ (m^2^). Nondominant middle arm circumference (MAC), waist and hip circumferences, and triceps skinfold thickness were also taken. Triceps muscle circumference (middle arm muscle circumference, MAMC) and area (middle arm muscle area, MAMA) were calculated.

Body composition was examined using a Bioelectrical Impedance Analyzer (BIA/STA, Akern, Florence, Italy). It is a distal, tetrapolar technique, delivering an excitation current at 50 kHz.

BIA gives two bioelectric parameters: body resistance (R) and reactance (Xc), and the impedance vector (Z) is a combination of R and Xc across tissues. The arc tangent of Xc/R is called phase angle (PA), which is a derived measure obtained from the relation between the direct measures of resistance and reactance reflecting hydration status and soft tissue cellular mass. Reduced phase angle reflects increased extra- to intracellular water ratio as well as a decrease in body cell mass. Body cell mass (BCM), skeletal muscle mass (SM) were derived from bio-impedance analysis and body cell mass index (BCMI) and skeletal mass index (SMI) were calculated [21].

### 2.3. Resting Energy Expenditure

Resting energy expenditure (REE) was measured by indirect calorimetry with a hand-held desktop calorimeter (Fitmate GS, Cosmed, Rome, Italy) using the dilution technique. The Fitmate’s accuracy has been validated against the gold standard Douglas Bag. The oxygen sensors were automatically calibrated before each measurement [22]. Patients arrived at 8:30 in the morning after 12 h fast. They were previously instructed not to exercise the day before the test. After their arrival, they were asked to rest for at least 20 min laying on a bed. Then, a ventilated hood (canopy) was placed over their head and shoulders making sure that the air did not enter from the outside or leak from the system. The canopy allows patients to breathe freely. The test lasted 20 min with measurements of oxygen consumption at 1 min intervals. REE was calculated from O^2^ consumption using a modified Weir equation [23].

### 2.4. Physical Activity and Performance

Spontaneous physical activity also was measured using the sense wear arm band (SWA, BodyMedia, Inc., Pittsburgh, PA, USA). The SenseWear armband is a multisensory body monitor and contains a two-axis accelerometer, a heat flux sensor, a galvanic skin response sensor, and skin and near-body temperature sensors. Subjects were asked to wear the armband on the upper arm for 24 h/day for 3 days and only remove it during periods when it might get wet (e.g., taking a shower or swimming). METs (metabolic equivalent of task) express the energy cost of physical activities and is defined as the ratio of metabolic rate during a specific physical activity to a reference metabolic rate, set by convention to 3.5 mL O_2_·kg^−1^·min^−1^.

### 2.5. Functional Tests

As physical performance tests, we performed the 30” Sit-to-Stand (30” STS) chair test, the six-minute walking test, and the Hand-grip.

The 30” sit-to-stand chair test (30” STS) is a validated test able to assess lower extremity strength in adults older than 60 years [24]. In the 30” STS chair test the participant is seated in the chair with his/her arms crossed and held against the shoulders. The score corresponds to the number of stands a person can complete in 30” without the help of arms. Data were compared also with standard values for age and gender.

The 6 min walking test (6MWT) was performed according to the American Thoracic Society guidelines [25]. This test measures the distance a subject is able to walk over a period of six minutes on a hard, flat surface. The goal is for the individual to walk as far as possible in six minutes. The subject walked along a 10 m path for a total of 22 m. The patient is allowed to self-pace and rest as needed [25].

The hand-grip was performed with a hydraulic hand Dinamometer ((Jamar, Sammons Preston Rolyan, Bolingbrook, IL, USA). Before this test was administered, the handle of the dynamometer was adjusted for the size of the subject. The handle should fit comfortably in the hand with enough allowance for a good grip. The subject was asked to place the arm at his side keeping it away from the body with the elbow bent slightly (we illustrate the use of the instrument to the subject prior to testing). The test was administered with dominant hand first and then with the nondominant hand. Emphasis on “squeeze as hard as you possibly can” and other forms of encouragement were used for maximum effect. We allow three trials with each hand, right and left hand alternately, with a brief pause of about 10 to 20 s between each trial to avoid excessive fatigue.

### 2.6. Statistical Analysis

Quantitative variables were described by mean ± standard deviation (SD) or median and interquartile range (IQR) when appropriate to compare quantitative variables and qualitative variables between groups *t*-test for unpaired data or Mann–Whitney test (when appropriate) and chi square test were applied respectively. Pearson’s correlation analysis was employed to determine associations between various selected quantitative parameters. Survival curves are calculated by Kaplan–Meier method and log-rank test was used to evaluate differences between curves. Finally, Cox regressions were performed both adjusted for and not adjusted for eGFR.

Statistical evaluation was carried out with IBM SPSS statistics v.25 (SPSS Inc., Chicago, IL, USA) for Windows. Differences were considered as statistically significant when *p* < 0.05.

## 3. Results

Table 1 shows the main clinical characteristics of the diabetic and nondiabetic CKD patients studied. The two groups did not differ for age, gender, residual renal function, albumin, hematocrit, bicarbonate, 25(OH)VitD, or electrolytes. Also, the prevalence of increased albumin excretion was not different in diabetic and nondiabetic (39% vs. 45%) CKD patients.

The use of diuretics was more prevalent in diabetic than in nondiabetic CKD patients (61% vs. 41%, *p* < 0.05), whereas no differences were observed for ACE-i (26% vs. 26%), ARB (43% vs. 51%), CCB (50% vs. 53%), beta-blockers (37% vs. 23%), or alpha1-blockers (13% vs. 11%), respectively.

Table 2 depicts the results of anthropometry, bioimpedance analysis, physical activity, and performance of the two groups. Diabetic patients have a higher body weight, BMI, waist circumference, fat mass, and a lower percentage of muscle mass than nondiabetic CKD patients.

The average daily METs values were significantly lower in diabetics CKD patients confirming a sedentary/low-activity life style. The time spent in activity >3 METs was similar in the two groups while the average number of steps per day was significantly higher in nondiabetic patients (Table 2).

The percentage of diabetic patients who showed a reduced handgrip strength (as expected according to age and sex) was similar to that of nondiabetic patients. Similarly, there was no difference in the 6MWT performance as well as no difference was found in the percentage of patients with a scarce performance capacity at the 6MWT (52.6% vs. 49.3%, *p* = 0.7401) [26,27].

Even though body weight and BMI were significantly higher in diabetics, no difference existed between diabetic and nondiabetic CKD patients with regards to the percentage of overweight/obesity on the basis of BMI (77% vs. 74.5%, *p* = 0.7322) and the percentage of FM, suggestive of overweight or obesity on the basis of WHO criteria (93% vs. 91%, *p* = 0.65606). Serum albumin levels were lower than 3.5 mg/dL only in the 5% of diabetic and 6% of nondiabetic CKD patients and hemoglobin levels were below 11 g/dL in 10.6% of diabetic and 5.3% of nondiabetic patients.

Measured resting energy expenditure was significantly higher in diabetics (Table 2), probably because of higher body weight and BMI. In fact, it was mitigated when normalized for body weight.

Energy intake normalized for body weight was lower in diabetics, but not significantly, and it was lower than that suggested by current guidelines. Even if low, energy intake is higher than daily energy expenditure derived from indirect calorimetry and METs and it is in line with the prevalence of overweight/obesity affecting the studied population. Protein intake, as estimated by calculation of protein catabolic rate, was similar in the two groups (Table 2) and in accordance with guidelines.

When all the CKD patients are included, the percentage of fat mass correlates negatively with 6MWT performance (r = −0.347, *p* < 0.0001), average daily walked distance (r = −0.35, *p* < 0.002) and correlates positively with resting energy expenditure (r = 0.245, *p* < 0.011). Dietary protein intake negatively correlates with MIS (r = −0.278, *p* < 0.012) and positively with average daily walked distance (r = 0.402, *p* < 0.001).

**Figure 1:**
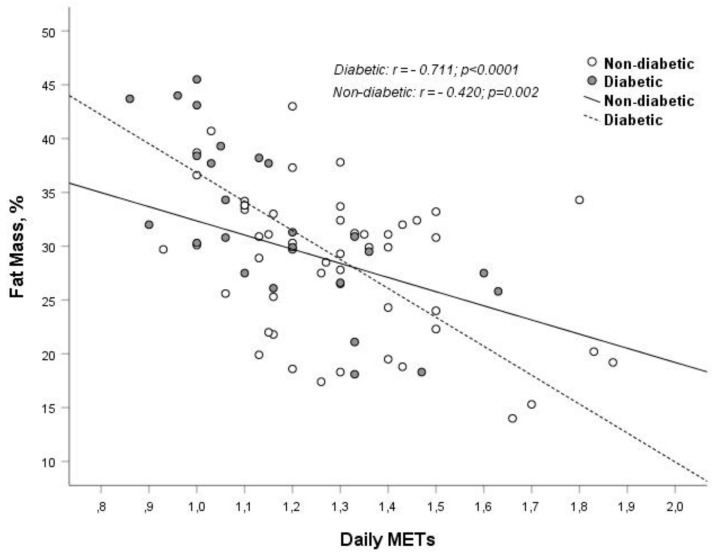
Correlation and linear regression analysis between daily METs and fat mass (%), stratified for diabetes.

Fat mass percentage negatively correlates with 6MWT performance (diabetics: r = −0.457, *p* < 0.006; no diabetic patients r = −0.257, *p* < 0.03) and with average daily METs (diabetics: r = −0.711, *p* < 0.0001; no diabetic patients r = −0.42, *p* < 0.002) (Figure 1). Performance at the 6MWT positively correlates with estimated protein intake normalized per body weight in diabetics (r = 0,423, *p* < 0.044) and nondiabetics (r = 0.401, *p* < 0.005). Waist circumference negatively correlates with average daily METs (r = −0.507, *p* < 0.008) in diabetics.

**Figure 2:**
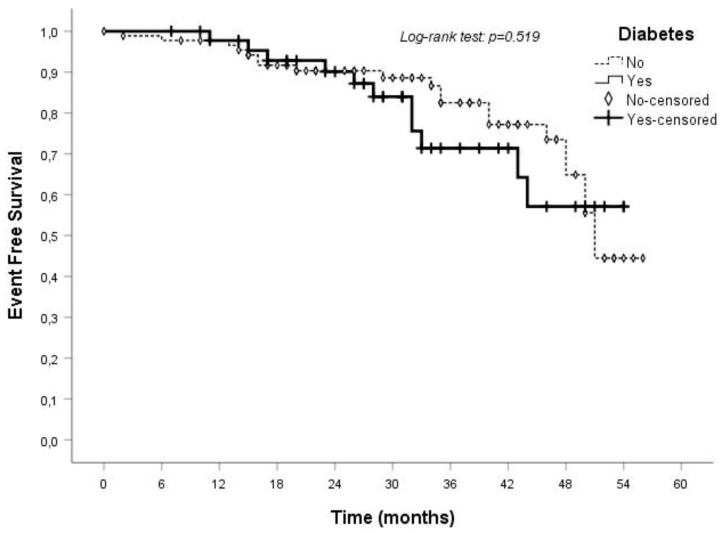
Event-free survival of diabetic and nondiabetic CKD patients.

During the follow-up period of 31 ± 14 months, the occurrence of death or dialysis commencing (or of doubling serum creatinine) was similar in the groups of diabetic and nondiabetic patients. Therefore, the presence of diabetes seems not to be associated with a greater risk for all-cause mortality or renal death in the studied CKD population on tertiary care management (Figure 2).

Table 3 reports the univariate correlation analysis (crude and eGFR-adjusted) for cumulative end-points (all-cause mortality or ESRD). The Cox regression adjusted for eGFR showed that middle arm muscle circumference, phase angle, serum cholesterol, and serum albumin were negatively related to the risk of events (Table 3). Each 1 cm increase of MAMC or each 0.2 g/dL increase of sAlbumin were associated with a 15% reduction of event risk; each one grade of phase angle was associated with 33% reduction of event risk, and each 10 mg/dL increase of total cholesterol was associated with a 20% decrease of event risk.

## 4. Discussion

Our findings show that at the same level of residual renal function, diabetic CKD patients have higher body weight and BMI due to increased abdominal and body fat mass associated with lower muscle mass, as compared to nondiabetic CKD patients. This occurs in the presence of similar REE and dietary energy intake but lower physical activity levels. The outcome of diabetic or nondiabetic CKD patients was similar in terms of risk for mortality or renal death. After normalization for residual renal function, only some nutritional and functional parameters remained predictive of poor outcome.

In diabetic patients, there are many mechanisms that can lead to an impairment of muscle strength and performance leading to reduced exercise capacity such as insulin resistance, hyperglycemia, muscle fat infiltration, peripheral neuropathies, and oxidative stress [28,29]. Several of these mechanisms exist in CKD patients because reduced kidney function leads to the retention of uremic solutes resulting in inflammation, oxidative stress and insulin resistance that promote skeletal muscle abnormalities. Diabetes is a condition contributing to further loss of muscle strength, because a decrease in skeletal muscle mass is a hallmark of kidney disease, and low physical activity adds limitations due to diminished condition.

Interestingly, if we compare the percentage of patients with a reduced physical capacity and performance at the 6MWT and the hand grip strength test, we found no difference in diabetic or nondiabetics patients. Therefore, the question is whether overweight/obesity and low physical capacity and performance is particular to diabetic CKD patients or a feature of the current CKD population and reflective of a poor lifestyle attitude.

The same happens when we compare the percentage of patients with higher BMI or FM, suggestive of overweight/obesity. We found no difference in diabetic or nondiabetic patients.

Overweight condition and obesity are notoriously associated with diabetes and in the last few years, the scientific literature also considers obesity as an important cause of kidney damage and chronic kidney disease [30,31]. Overweight condition and obesity are associated with hemodynamic, structural and histopathological alterations in the kidney, as well as metabolic and biochemical alterations that predispose one to kidney disease (even when renal function is normal).

It is noteworthy that the high percentage of overweight/obesity and reduced physical activity and capacity is not limited to the diabetic cohort.

The analysis of outcome of the studied population shows that body composition and physical performance abnormalities are the most important predictors of mortality or renal death in our studied cohorts. A similar outcome between diabetic and nondiabetic patients was observed, as well. The literature reports worse outcomes in diabetic than in nondiabetic CKD patients, but Piccoli et al. [32] reported that in the last CKD stages, and under the effect of a moderately restricted low-protein diet, the risk of death and of progressing to dialysis is comparable to that of nondiabetic subjects, after adjusting for comorbidity. Our results are in keeping with those of Piccoli et al. and it underlines the importance of tertiary care management, including nutritional therapy, particularly in diabetic CKD patients. The high prevalence of nonproteinuric phenotype [33] can contribute to explain this finding.

Body composition, physical activity, and physical function are strictly and reciprocally linked to one another: a reduction in physical activity causes a worsening in physical function with the latter more consistently predicting declines of physical activity [34].

Obesity and overweight state, even in the condition of metabolic health, accelerate age-related declines in functional ability and threaten independence in older age. High fat percentage and reduced muscle mass may limit basic physical actions (climbing stairs, walking) and everyday life activities, thus favoring further muscle impairment and weight gain. Therefore, we have a negative reinforcement loop that perpetuates itself.

Recently Dong et al. reported that lifestyle changes including exercise (namely at least 300 min of physical activity similar to brisk walking) and diet reduce the inflammatory state, delaying renal fibrosis and the development of renal failure, thus reducing the risk of diabetic nephropathy progression [35].

Vitamin D status is another important nutritional aspect of CKD patients, particularly in diabetics who are higher risk of vitamin D deficiency. In our study, 25(OH)VitD serum levels were similar in diabetic and nondiabetic CKD patients, and we did not find any relationship between 25(OH)VitD serum levels and renal outcome. In addition, in our series, 25(OH)VitD serum levels below 23 ng/mL were not associated with CKD progression. This is not in keeping with a previous study [36], and it could be due to the lower number of patients, and by the tertiary care management that included treatment of Vitamin D deficiency, if any.

## 5. Conclusions

In summary, this study confirms that diabetic CKD patients have higher body weight and BMI with increased abdominal and body fat mass associated with lower muscle mass with respect to nondiabetic CKD patients. This occurs in the presence of similar REE and dietary energy intake but lower physical activity levels. The outcome of diabetic or nondiabetic CKD patients on tertiary care management was similar in terms of risk for mortality or renal death. Given the same residual renal function, low levels of muscle mass, phase angle, serum albumin, and cholesterol were associated with increased risk of events. Overall, a malnutrition phenotype represents a major predictor of poor outcome in diabetic and nondiabetic CKD patients.

This suggests that a routine nutritional evaluation and assessment of physical activity and capacity may have a role in the care management of CKD patients. In particular, type 2 diabetic CKD patients should receive special attention to couple a correct nutritional plan along with implementation of physical activity programs.

## Figures and Tables

**Table 1:** Clinical characteristics of diabetic and nondiabetic chronic kidney disease (CKD) patients. Mean ± SD or median (IQR).

	Diabetic CKD	Nondiabetic CKD	*p* Value
Gender, m/f	40/8	80/16	
Age, yrs	71.5 ± 8.2	71.6 ± 9	0.875
eGFR, ml/min×1.73 m^2^	29.8 ± 9.1	31.3 ± 10.9	0.398
BUN, mg/dL	43 ± 16	37.6 ± 12.2	0.044
sCreatinine, mg/dL	2.26 (1.77–2.78)	2.10 (1.68–2.82)	0.596
sSodium, mEq/L	141 (140–142)	140 (139–142)	0.843
sPotassium, mEq/L	4.78 ± 0.52	4.70 ± 0.52	0.406
sCalcium, mg/dL	9.33 ± 0.41	9.29 ± 0.48	0.602
sPhosphate, mg/dL	3.34 ± 0.63	3.33 ± 0.56	0.915
Bicarbonate, mEq/L	25.4 ± 3.7	24.7 ± 2.7	0.354
sTotal Protein, g/dL	7.10 ± 0.48	7.14 ± 0.47	0.614
sAlbumin, g/dL	4.18 ± 0.45	4.16 ± 0.38	0.762
Hemoglobin, g/dL	13.1 ± 1.7	13.2 ± 1.6	0.743
Hematocrit, %	38.8 ± 5.8	39.7 ± 4.5	0.332
25(OH)VitD, ng/mL	20.5 ± 10.3	22.4 ± 10.9	0.398
sCholesterol, mg/dL	170 ± 38	179 ± 39	0.197

**Table 2:** Anthropometry, bioimpedance analysis, physical activity, and performance parameters in diabetic and nondiabetic CKD patients.

	Diabetic CKD *n* = 48	Nondiabetic CKD *n* = 96	*p* Value
Body weight, kg	83.1 ± 15	76.4 ± 12.3	0.0090
BMI, kg/m^2^	29.1 ± 4.64	27.2 ± 3.52	0.0144
Waist circ., cm	104.7 ± 11.9	99.4 ± 12.4	0.0162
MAMC, cm	26.0 ± 4.2	25.3 ± 10	0.5581
MAMA, cm^2^	55.4 ± 16.9	58.9 ± 86.5	0.7030
Phase angle, °	5 ± 1	4.9 ± 1	0.8655
BCMI, kg/m^2^	9.5 ± 1.9	9.4 ± 2.9	0.8423
FM, %	31.5 ± 7.4	27.8 ± 6.7	0.0071
MM, %	40.1 ± 5.3	43.2 ± 5.7	0.0324
Average daily Mets	1.17 ± 0.2	1.29 ± 0.2	0.0167
Average daily steps	3580 ± 2471	5628 ± 4143	0.0080
6MWT, m	318 ± 94	331 ± 84	0.4839
Hand grip, kg	29.1 ± 7.5	26.5 ± 7.9	0.0602
REE, kcal/d	1341 (1231–1531)	1224 (1150–1468)	0.012
nPCR, g/kg b.w/d	0.86 ± 0.22	0.81 ± 0.19	0.3481

Statistics: mean ± SD or median (IQR). MAMC: middle arm muscle circumference; MAMA: middle arm muscle area; BCMI: body cell mass index; FM: fat mass; MM: muscle mass; MET: metabolic equivalent task; PA: physical activity; STS30”: sit to stand 30”; 6MWT: six-minute walking test; REE: resting energy expenditure; nPCR: normalized protein catabolic rate. (Mean ± SD or median (IQR)).

**Table 3:** Univariate correlation analysis (crude and eGFR-adjusted) for cumulative end-points (all-cause mortality or ESRD). MAMC: middle arm muscle circumference; BCM: body cell mass; FFM: free fat mass; nPCR: normalized protein catabolic rate; 6MWT: six-minute walking test.

	Univariate Analysis		eGFR Adjusted
	HR (CI 95%)	*p* Value	*p* Value
Diabetes, (0) no; (1) yes	1.26 (0.62–2.57)	0.691	0.414
MAMC, cm	0.85 (0.78–0.91)	<0.001	<0.001
Phase angle, °	0.67 (0.46–0.98)	0.041	0.025
BCM, kg	0.95 (0.89–1.01)	0.125	0.184
FFM, kg	0.99 (0.95–1.02)	0.475	0.963
Urea, mg/dL	1.03 (1.02–1.04)	<0.001	0.088
Phosphate, mg/dL	3.20 (1.61–6.34)	0.001	0.082
Total cholesterol, mg/dL	0.98 (0.97–0.99)	0.001	<0.001
sAlbumin, g/dL	0.24 (0.09–0.66)	0.005	0.024
Hemoglobin, g/dL	0.66 (0.51–0.86)	0.002	0.072
Hematocrit, %	0.95 (0.90–0.97)	0.034	0.221
nPCR*, g/kg b.w/d	0.13 (0.01–1.55)	0.106	0.768
6MWT, m	0.98 (0.99–1.01)	0.203	0.373
